# Unified Transcriptomic Signature of Arbuscular Mycorrhiza Colonization in Roots of *Medicago truncatula* by Integration of Machine Learning, Promoter Analysis, and Direct Merging Meta-Analysis

**DOI:** 10.3389/fpls.2018.01550

**Published:** 2018-11-12

**Authors:** Manijeh Mohammadi-Dehcheshmeh, Ali Niazi, Mansour Ebrahimi, Mohammadreza Tahsili, Zahra Nurollah, Reyhaneh Ebrahimi Khaksefid, Mahdi Ebrahimi, Esmaeil Ebrahimie

**Affiliations:** ^1^Australian Centre for Antimicrobial Resistance Ecology, School of Animal and Veterinary Sciences, The University of Adelaide, Adelaide, SA, Australia; ^2^Institute of Biotechnology, Shiraz University, Shiraz, Iran; ^3^Department of Biology, University of Qom, Qom, Iran; ^4^Department of Biotechnology, Shahrekord University, Shahrekord, Iran; ^5^School of Agriculture Food and Wine, Department of Plant Science, The University of Adelaide, Adelaide, SA, Australia; ^6^Max-Planck-Institute for Informatics, Saarbrucken, Germany; ^7^Adelaide Medical School, The University of Adelaide, Adelaide, SA, Australia; ^8^Division of Information Technology, Engineering and the Environment, School of Information Technology and Mathematical Sciences, University of South Australia, Adelaide, SA, Australia; ^9^Faculty of Science and Engineering, School of Biological Sciences, Flinders University, Adelaide, SA, Australia

**Keywords:** machine learning, meta-analysis, regulatory mechanism, symbiosis, systems biology

## Abstract

Plant root symbiosis with Arbuscular mycorrhizal (AM) fungi improves uptake of water and mineral nutrients, improving plant development under stressful conditions. Unraveling the unified transcriptomic signature of a successful colonization provides a better understanding of symbiosis. We developed a framework for finding the transcriptomic signature of Arbuscular mycorrhiza colonization and its regulating transcription factors in roots of *Medicago truncatula*. Expression profiles of roots in response to AM species were collected from four separate studies and were combined by direct merging meta-analysis. Batch effect, the major concern in expression meta-analysis, was reduced by three normalization steps: Robust Multi-array Average algorithm, Z-standardization, and quartiling normalization. Then, expression profile of 33685 genes in 18 root samples of *Medicago* as numerical features, as well as study ID and Arbuscular mycorrhiza type as categorical features, were mined by seven models: RELIEF, UNCERTAINTY, GINI INDEX, Chi Squared, RULE, INFO GAIN, and INFO GAIN RATIO. In total, 73 genes selected by machine learning models were up-regulated in response to AM (Z-value difference > 0.5). Feature weighting models also documented that this signature is independent from study (batch) effect. The AM inoculation signature obtained was able to differentiate efficiently between AM inoculated and non-inoculated samples. The AP2 domain class transcription factor, GRAS family transcription factors, and cyclin-dependent kinase were among the highly expressed meta-genes identified in the signature. We found high correspondence between the AM colonization signature obtained in this study and independent RNA-seq experiments on AM colonization, validating the repeatability of the colonization signature. Promoter analysis of upregulated genes in the transcriptomic signature led to the key regulators of AM colonization, including the essential transcription factors for endosymbiosis establishment and development such as *NF-YA* factors. The approach developed in this study offers three distinct novel features: (I) it improves direct merging meta-analysis by integrating supervised machine learning models and normalization steps to reduce study-specific batch effects; (II) seven attribute weighting models assessed the suitability of each gene for the transcriptomic signature which contributes to robustness of the signature (III) the approach is justifiable, easy to apply, and useful in practice. Our integrative framework of meta-analysis, promoter analysis, and machine learning provides a foundation to reveal the transcriptomic signature and regulatory circuits governing Arbuscular mycorrhizal symbiosis and is transferable to the other biological settings.

## Introduction

Arbuscular mycorrhiza (AM) fungal symbiosis expands the surface area of plant root, allowing for better absorption of substances such as phosphorus, ammonium, and zinc from soil. This symbiosis supports plant development, particularly under nutrient deficiency and other stressful conditions. Specific genetic programs activated by AM inoculation lead to successful microsymbiont colonization and functional symbiosis. Most studies in AM symbiosis are limited to the investigation of a single gene or a cluster of similar genes. Genes such as *DMI1, DMI2, NFP, NSP1* (Oláh et al., 2005), *MtBcp1* (Hohnjec et al., 2005), *ENOD11* (Genre et al., 2005), *MIG1* (Heck et al., 2016), RAM1 (Rich et al., 2017), *nfr1, nfr5*, l*ys11* (Rasmussen et al., 2016), and *NIN* (Guillotin et al., 2016) are reported to play roles in the formation of mycorrhizal symbiosis.

The regulatory mechanisms underpinning AM symbiosis in plants are poorly understood. The GRAS transcription factor family contains the best known regulators of AM symbiosis. The function of *ATA/RAM1*, a member of this family, in reprogramming AM symbiosis has been established (Rich et al., 2017). It has been suggested that *RAM1* controls the expression of many essential AM-related genes such as *STR, STR2, RAM2*, and *PT4* (Rich et al., 2017). Another member of the GRAS transcription factor family, *MIG1*, interacts with *DELLA1* and the root GA signaling pathway to regulate cortical cell expansion in developing AM symbiosis (Heck et al., 2016). The role of small RNAs, such as *miR171* in establishment of AM symbiosis has also been investigated recently (Couzigou et al., 2017).

Successful AM colonization is vital to establish symbiosis and improve phosphorous and water uptake. The AM type, as well as many, environmental and genetic factors affect the intensity, timing, and the success of AM colonization. Cross-comparison of successful colonization between different AM types in a range of experiments by meta-analysis provides the opportunity to move toward understanding the genetic basis of endosymbiosis (Tromas et al., 2012), the conserved transcriptomic program that can reflect successful AM colonization and establishment. Those genes can unravel the functional groups that may play key roles in the establishment and functioning of the three AM symbioses. The transcriptomic signature of AM colonization can be further employed for: (1) increasing AM efficiency by application of chemical and environmental treatments, (2) monitoring successful/unsuccessful AM colonization, and (3) finding the upstream regulatory mechanisms and regulators such as transcription factors and microRNAs that control AM colonization and symbiosis.

However, no attempt has been made to identify the unified transcriptomic signature of AM symbiosis. The term of “Unified transcriptomic signature” or “biosignature” refers to robust transcript responses that can monitor the successful AM colonization. Overlaps observed in transcriptional profiles of *Medicago truncatula* roots inoculated with two different *Glomus* fungi (Hohnjec et al., 2005) support the possibility of achieving a unified transcriptomic signature of AM colonization to provide an insight into the genetic program activated during AM.

The emerging field of meta-analysis may solve the issue of merging different experiments to identify a unique biosignature of Medicago root response to AM inoculation. Cross-species meta-analysis of transcriptomic data has received increased attention in recent years due to the advances in pattern discovery and meta-analysis models (Tromas et al., 2012; Farhadian et al., 2018b). Meta-analysis enables the combination of expression datasets and is highly advantageous in increasing statistical power to detect biological phenomena from studies with a restricted sample size (Johnson et al., 2007).

The biosignature of AM inoculation obtained may be utilized to further computational systems biology analysis, such as promoter analysis, common regulator discovery, and common target discovery, in order to lead us to the key regulators and targets of the AM symbiosis pathway.

Different statistical methods have been developed for meta-analysis of expression data such as combining effect sizes, combining ranks, combining *p*-values, vote counting, and direct merging (DM) (Borenstein et al., 2009, 2010; Campain and Yang, 2010; Chang et al., 2013; Sharifi et al., 2018). Within meta-analysis approaches, DM analysis of expression data or genomic variant data of different studies is an attractive meta-analysis method to increase statistical power and lead to a robust transcriptomic or genomic signature (Tseng et al., 2012). DM, as a meta-analysis approach, has been used in web-tools such as INMEX (Xia et al., 2013, 2015), A-MADMAN (Bisognin et al., 2009), WGAS (Dai et al., 2007), and GEOSS (Bisognin et al., 2009) for integrative meta-analysis of expression data. DM-based meta-analysis provides the possibility of data collection from different experiments, even when a treatment or a control is missing in one or more experiments. This contributes to a higher statistical power of meta-analysis.

The major concern about the DM approach is heterogenicity across studies. The success of the DM approach depends on normalization across studies to reduce non-biological experimental variation as well as biological variations unrelated to treatment (also called batch effects or study effects) (Johnson et al., 2007; Tseng et al., 2012). Collection of arrays from similar platforms across all studies (mainly Affymetrix) and pre-processing of the CEL expression files by model-based robust multi-array (RMA) normalization (Irizarry et al., 2003) have been suggested to decrease heterogenicity across all studies (Lee et al., 2008; Sims et al., 2008; Tseng et al., 2012). However, it has been debated that RMA is not strong enough to remove batch effects (Guerra and Goldstein, 2009). To sufficiently reduce batch effects for accurate DM, additional normalization techniques such as empirical Bayes methods (Johnson et al., 2007), cross-platform normalization (Shabalin et al., 2008), weighted distance weighted discrimination (Qiao et al., 2010), enrichment-based meta-analysis, and Ratio adjustment and calibration scheme (Cheng et al., 2009) have been used.

Recent advances in application of supervised machine learning models in transcriptomic studies have opened a new venue to engage data mining models in decreasing batch effects and integration of different studies (Pashaiasl et al., 2016a,b). Supervised machine learning has brought new possibilities to predictive studies (Bakhtiarizadeh et al., 2014a; Ebrahimi et al., 2014; Zinati et al., 2014; Kargarfard et al., 2015; Pashaiasl et al., 2016a,b). The capability to simultaneously analyse both categorical and numerical features, power to analyse large data, and various predictive algorithms with diverse statistical backgrounds are distinguished features of supervised machine learning models (Shekoofa et al., 2014; Ebrahimi et al., 2015; Jamali et al., 2016). The possibility to include the categorical variables in predictive models can outstandingly decrease the heterogenicity across studies as the batch effects (Shekoofa et al., 2014). For example, in this study, the different experiments or types of AM can be added as variables and analyzed in the predictive model of the AM transcriptomic signature. This possibility is highly limited in traditional multivariate or regression models.

Due to the central role of colonization in establishing a microsymbiont, we developed a framework for finding the transcriptomic signature of successful AM colonization on roots of *Medicago truncatula* by integration of meta-analysis and machine learning (attribute weighting) models. Special attention was paid to reducing the batch effects by utilizing normalization methods and finding reliable gene candidates by machine learning models. The genes discovered in the transcriptomic signature were further used as the input of promoter analysis to identify the transcription factors which regulate the signature.

## Methods

**Figure 1:**
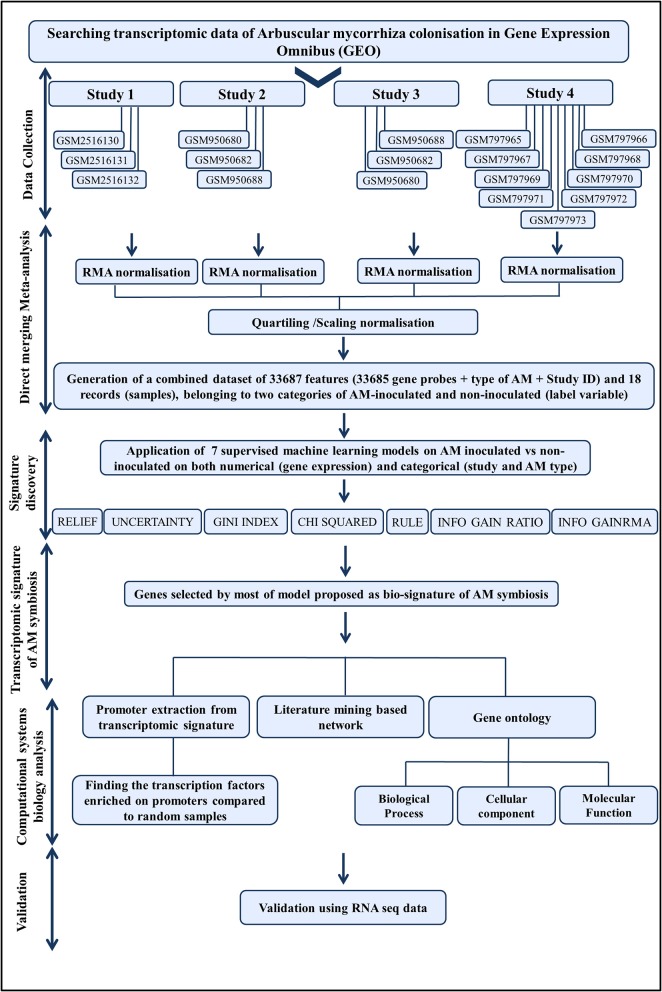
The flowchart of computational systems biological approach, developed in this study.

A flowchart of the integrative computational systems biological approach employed in this study is presented in Figure 1.

### Data collection for meta-analysis

Studies on the AM transcriptome were identified in repositories of high-throughput expression data such as NCBI GEO (https://www.ncbi.nlm.nih.gov/geo/) and ArrayExpress (https://www.ebi.ac.uk/arrayexpress/). Supplementary Table 1 presents the list of the studies mined and their platforms. The Microarray studies belonged to *Medicago truncatula* A17.

The microarray experiment (Floss et al., 2017) was originally designed to compare gene expression in roots of *Medicago truncatula* A17 and *Medicago truncatula* mutant mtpt4-1 colonized with *Gigaspora gigantea*. We only used data from three independent biological replicate samples of wild type plants colonized with *Gigaspora gigantea* for meta-analysis. Samples were harvested 18-day post planting and 11 days post contact with the spores.

In the original experiment (Truong et al., 2015), the impact of P limitation and both P and N limitation on *Medicago truncatula* A17 root transcriptome in response to *Rhizophagus irregularis* (previously known as *Glomus intraradices*) were investigated. In the original experiment, the root transcriptome of both wild type plants and a hypermycorrhizal mutant (B9) grown on limiting or non-limiting phosphate were analyzed to determine which processes were in the hypermycorrhizal mutant. Plants were harvested 4 weeks after inoculation. From this experiment, only data of mycorrhizied wild type plants colonized with *Rhizophagus irregularis* and grown under P limitation were used for our meta-analysis study.

In the experiment of Hogekamp et al. (2011), gene expression profiles of roots of *Medicago truncatula* A17 in response to colonization by two different arbuscular mycorrhizal fungi (*Rhizophagus irregularis* and *Glomus mosseae*) as well as P treatment with phosphate were studied. From this experiment, data of two groups of samples were used for meta-analysis; data of inoculated plants and non-inoculated plants under P limitation. Non-inoculated plants were used as control.

CEL (expression intensity) files of these studies were downloaded from NCBI GEO databank and their corresponding library (CDF) and annotation (CSV) files from the Affymetrix FTP repository by Affymetrix Expression Console Software (version: 1.3.1.187, https://www.affymetrix.com/).

### Reducing the batch effect in direct merging (DM) meta-analysis

Reducing heterogenicity across studies (batch effects) is an essential step for direct combination of expression data in DE meta-analysis. Here, we developed an integrative approach including multi-array (RMA) normalization within studies, Z-standardization of expression values, and between studies quartiling/scaling normalization for reducing batch effects before combining samples for supervised machine learning.

#### Rma normalization of samples in each study (within study normalization)

CEL files of Affymetrix arrays in each study were normalized by an RMA algorithm (Irizarry et al., 2003) using Affymetrix Expression Console Software (version: 1.3.1.187).

#### Z-value standardization

Z-standardization has been extensively used in meta-analysis (Lipsey and Wilson, 2001; Kinoshita and Obayashi, 2009). RMA normalized expression values of each samples were converted to Z-value by subtracting the mean and dividing by the standard deviation using Minitab 17 (www.minitab.com/).

#### Between sample normalization by scaling and quartiling

To unify the RMA-normalized and Z-standardized values, we used an additional normalization step. Here, we evaluated the efficiency of the scaling and quartiling approach (Bolstad et al., 2003) in reducing the batch effects, using CLC Genomics Workbench (QIAGEN, https://www.qiagenbioinformatics.com/products/clc-genomics-workbench/). Supplementary Figure 1 shows the pseudo code for scaling and quartiling approach. In the Scaling approach (Supplementary Figure 1A), the sets of the expression values for the samples were multiplied by a constant so that the sets of normalized values for the samples have the same ‘target' value. The target (normalization) value was defined as Median mean/Median median of all samples. The Mean and Median are the types of normalization value of the samples to ensure that they are equal for the normalized expression values.

In quartiling approach (Supplementary Figure 1B), the empirical distributions of the sets of expression values for the samples were used to calculate a common target distribution, which was used to calculate normalized sets of expression values for the samples. Here, the term of empirical distribution refers to real (empirical) statistical characteristics of samples to be used for calculation of normalization values.

### Application of seven supervised machine learning models to find the medicago response genes distinguishing AM colonized from non-colonized symbiosis

At first, a cleaning step was performed and the probsets with no gene annotation, or the ones which matched to multiple genes were removed. Then, the expressions of 33685 probsets/genes in AM colonization and non-colonization conditions, as numerical features, were mined by seven attribute weighting (feature selection) models. Also, study number and type of AM (*Gigaspora gigantean, Rhizophagus irregularis, Glomus mosseae*, or none) were added to the dataset as categorical features. Consequently, a dataset of 33687 (33685 gene probes + type of AM + Study ID) and 18 records (samples), belonging to two categories of AM-inoculated and non-inoculated (label variable), were used for machine learning. The selected feature selection models were able to analyse both categorical and numerical features simultaneously. This provided the opportunity to assess batch effects.

Feature selection models identify the most important genes whose AM expression differs between colonized and non-colonized symbioses. The resulting weights of each feature selection model were normalized into the interval between 0 and 1 to provide the similar significance across various feature selection models. Weights closer to 1 show a higher relevance (importance) of a particular gene in distinguishing AM inoculated from non-inoculated roots, according the employed feature selection model. The genes determined to be important by most of the feature selection models (intersection of weighting methods with various statistical backgrounds) with cut-off ≥ 0.95 were assumed to be the key distinguishing genes to form the biosignature. The employed feature selection models were: RELIEF, UNCERTAINTY, GINI INDEX, CHI SQUARED, RULE, INFO GAIN RATIO, and INFO GAIN.

RELIEF is a classification attribute weighting model, independent from Heuristic search and is considered to be one of the most successful models for evaluating the quality of features because of its simplicity and efficiency. RELIEF is a robust noise-tolerant model able to feature interactions where it employs the random selection of instances for weight estimation (Kira and Rendell, 1992; Rosario and Thangadurai, 2015). RELIEF estimates the relevance of attributes (genes + study number + AM type) according to how well their values discriminate between the instances of the same and different classes of label (AM colonization/non-colonization) that are near each other (Ebrahimi et al., 2014).

UNCERTAINTY measures the weight of attributes (genes + study number + AM type) against the label attribute (AM colonization/non-colonization) by estimating the symmetrical uncertainty with respect to the class (Liang, 2011).

GINI INDEX attribute weighting algorithm evaluates the weight of attributes (genes + study number + AM type) by computing the Gini index of the class distribution (AM colonization/non-colonization) and is a measure of data impurity (Lerman and Yitzhaki, 1984; Ebrahimi et al., 2011).

CHI SQUARED attribute weighting model evaluates the importance of attributes (genes + study number + AM type) with respect to the label attribute (AM colonization/non-colonization) based on chi squared statistic (Ebrahimi et al., 2014).

INFO GAIN model calculates the relevance of attributes (genes + study number + AM type) by measuring the Information Gain in class distribution (AM colonization/non-colonization) (Guyon and Elisseeff, 2003). INFO GAIN is suitable for datasets such as the expression of genes where attributes cannot take a large number of distinct values.

INFO GAIN RATIO uses information Gain Ratio for feature selection. This model is a modified version of INFO GAIN that biases against considering attributes with a large number of distinct values (Zinati et al., 2014).

RULE attribute weighting model estimates the weights of attributes (genes + study number + AM type) with respect to the label attribute (AM colonization/non-colonization) by constructing a single rule for each attribute and calculating the error (Liu and Motoda, 2012).

### Multivariate analysis of the developed AM transcriptomic signature

After developing the AM transcriptomic signature by integration of meta-analysis and machine learning, clustering based on the Average Linkage method and Euclidean distance measure, as well as cross validation based on Discriminant (modeling) analysis, was used to evaluate the power of the emergent AM transcriptomic signature for discrimination of AM-inoculated from non-inoculated samples. For clustering, the expression values of genes which formed the transcriptome biosignatures were standardized. The multivariate analyses mentioned were performed using Minitab 17 (www.minitab.com/).

Based on the paper published by Hogekamp et al. (2011), we also investigated the fitness of some previously-reported markers of the mycorrhizal symbiosis, including MtLec5 (legume lectin family protein, *MTR_5g031030*), MtGIP1 (germin-like protein 9-2, *MTR_4g052770*), MtPt4 (high affinity inorganic phosphate transporter, *MTR_1g028600*), and MtBcp1 (blue copper-like protein, MTR_6g013420).

### Gene ontology (GO) analysis

For a better understanding of the biological importance of the identified AM transcriptomic signature, we used a Gene Ontology (GO) approach that classifies genes and proteins based on a controlled functional vocabulary in terms of their Molecular Function, Biological Process, and Cellular Component (Ashburner et al., 2000; Fruzangohar et al., 2013, 2017). Unregulated genes (73 in total) in the AM inoculation transcriptomic signature with a Z-value difference of >0.5, were announced important by most feature selection models that were used as input of Ensembl Biomart and agriGO web applications (Kinsella et al., 2011; Tian et al., 2017). Agrigo employs the Fisher test and FDR correction for identifying the significance of GO terms of input genes compared to whole genome GO distribution (as a control/background).

### Upstream regulatory (common TFs) analysis of AM colonization signature through promoter analysis of highly expressed meta-genes in response to AM inoculation

The developed transcriptomic signature of successful AM colonization of roots of *Medicago truncatula*, obtained by integration of meta-analysis and machine learning (attribute weighting) models, was used for upstream regulatory analysis through common regulator discovery, as previously described (Deihimi et al., 2012; Babgohari et al., 2014; Bakhtiarizadeh et al., 2014b; Shamloo-Dashtpagerdi et al., 2015). To this end, the top 20 highly upregulated meta-genes, which responded to AM inoculation, (Supplementary Table 5) were selected for promoter analysis and common transcription factor discovery. In other words, the genes revealed in the transcriptomic signature were further used as the input of promoter analysis to find the transcription factor matrix families which regulate the transcriptome signature. Matrix families are groups of weight matrices for the same or functionally similar transcription factors (Cartharius et al., 2005).

To mine the binding of transcription factor families to the promoter regions, we used the MatInspector webtool (Quandt et al., 1995; Cartharius, 2005; Cartharius et al., 2005; Hosseinpour et al., 2013) to calculate the following scores: Core similarity, Matrix similarity, Model similarity, Free energy, Match rate, and *p*-value for the common TFs. Core similarity describes the similarity between core sequence of transcription factor matrix family and the input sequence. Core sequence of a transcription factor matrix family is the consecutive highest conserved positions of the matrix. The maximum core similarity of 1.0 is only reached when the highest conserved bases of a matrix match exactly with the input sequence. Matrix similarity is more important than the core similarity that takes into account all bases over the whole matrix length. Matrix similarity of 1.0 reaches only if the candidate sequence corresponds to the most conserved nucleotide at each position of the matrix. The free energy (in kcal/mol) is a thermodynamic parameter for the stability of secondary structures (hairpins) of matrix family with input sequence. The higher the free energy, the more stable the hairpin is. The match rate is the number of matching base pairs in percent of the total element length. The *p*-value for the common TFs is the probability to obtain an equal or greater number of sequences with a match in a randomly drawn sample of the same size as the input sequence set using Fisher's exact test. The lower this probability, the higher the importance of the observed common transcription factors.

Based on *p* < 0.01 of the common TFs and Matrix similarity >0.95, the enriched transcription factors on promoter regions of AM colonization signature were recorded as “common regulators.” In other words, transcription factors with the highest number of possible interactions with the upregulated genes after AM inoculation were assumed as the key regulators, called common regulators of AM inoculation.

### Independent validation of meta-genes based biosignature of AM inoculation by RNA-Seq

For independent validation of the AM colonization signature, derived by integration of meta-analysis and supervised attribute weighting models, independent samples of AM-inoculated and non-inoculated from RNA-seq experiment with GEO accession of GSE94266 (Garcia et al., 2017) were selected. The original experiment was designed to determine the effect of K^+^ on colonization of *Medicago truncatula* plants (Garcia et al., 2017). Plants were co-cultured with the AM fungus *Rhizophagus irregularis* under normal and low K+ regimes. We used 3 AM-inoculated samples (GEO accessions: GSM2471944, GSM2471945, and GSM2471946) and 3 AM non-inoculated samples (GSM2471950, GSM2471951, and GSM2471951) of this experiment under normal K^+^ regime to investigate the transcriptome response of *Medicago truncatula* to AM inoculation. Raw SRA files of the above-mentioned samples (100 bp, single end, Illumina sequencing technology) were retrieved by the DRAsearch tool (http://trace.ddbj.nig.ac.jp/DRASearch/) of the Research Organization of Information and System National Institute of Genetics (NIG), Japan. SRA files were transformed to fastq files using SRA Toolkit software (NCBI).

The *Medicago truncatula* reference genome (Mt4. 0v2 Assembly), including fasta (genome sequence) and GFF3 (genome annotation) files, were downloaded from the *Medicago truncatula* Genome Database (Young et al., 2011; Krishnakumar et al., 2014). Quality control of reads was analyzed using FastQC package (https://www.bioinformatics.babraham.ac.uk/projects/fastqc/). Low quality reads and adaptor sequences were trimmed by the CLC Genomics Workbench 11.0.1 (QIAGEN). Mapping of short reads to the reference genome was performed using the CLC Genomics Workbench using the following criteria: mismatch cost = 2, insertion cost = 3, deletion cost = 3, length fraction = 80%, and similarity fraction = 80%). Total counts of mapped reads and RPKM (Reads Per Kilobase of transcript per Million mapped reads) were recorded as expression measurements for each gene. The differences related to the depth of sequencing were corrected by per-sample library size normalization using TMM (trimmed mean of M values) normalization method via calculating and adjusting the effective libraries sizes (Robinson and Oshlack, 2010).

To find the differentially expressed genes during AM-colonization vs. non-colonization, Generalized Linear Model (GLM) based on Negative Binomial distribution (Anders and Huber, 2010) was used to fit curves to expression values without assuming that the error on the values is normally distributed. GLM-based *p*-values for differentially expressed genes were calculated and corrected using FDR statistics and the CLC Genomics Workbench tool. Also, fold changes were calculated from the GLM to correct for differences in library size between the samples and the effects of confounding factors.

To visualize the differentially-expressed genes by heatmap, the following steps were performed: (1) log CPM (Counts per Million) values were calculated for each gene. The CPM calculation uses the effective library size, calculated by the TMM normalization. (2) log CPM values were standardized across samples for each gene by transforming to *Z*-values.

## Results

### Selected samples from different studies for de meta-analysis

As the transcriptomic signature of AM may differ in different tissues, we selected the transcriptome files of root samples of *Medicago truncatula* A17. To reduce the variation between experiments, the *Medicago* Genome Array of Affymetrix platform with 61278 probset IDs was selected. Some samples in four studies had these criteria (Table 1, Figure 1) (Hogekamp et al., 2011; Bonneau et al., 2013; Truong et al., 2015; Floss et al., 2017). These samples were roots colonized with *Gigaspora gigantean, Rhizophagus irregularis*, or *Glomus mosseae*as well as non-inoculated ones.

**Table 1:** The studies and samples used in this study for obtaining the unified transcriptomic signature of *Arbuscular mycorrhiza* in roots of *Medicago truncatula*.

**Study**	**Reference**	**Sample**	**GEO accession of experiment**	**Strain of arbuscular mycorrhiza**	**Treatment**	**Platform**	**Type of platform**	**GEO accession of sample**
1	PMID: 28392110	1	GSE95545	Gigaspora gigantea	AM inoculated	Affymetrix	Medicago Genome Array	GSM2516130
1	PMID: 28392110	2	GSE95545	Gigaspora gigantea	AM inoculated	Affymetrix	Medicago Genome Array	GSM2516131
1	PMID: 28392110	3	GSE95545	Gigaspora gigantea	AM inoculated	Affymetrix	Medicago Genome Array	GSM2516132
2	PMID: 23506613	4	GSE38847	Rhizophagus irregularis	AM inoculated	Affymetrix	Medicago Genome Array	GSM950680
2	PMID: 23506613	5	GSE38847	Rhizophagus irregularis	AM inoculated	Affymetrix	Medicago Genome Array	GSM950682
2	PMID: 23506613	6	GSE38847	Rhizophagus irregularis	AM inoculated	Affymetrix	Medicago Genome Array	GSM950688
3	PMID: 24815324	7	GSE44102	Rhizophagus irregularis	AM inoculated	Affymetrix	Medicago Genome Array	GSM1078957
3	PMID: 24815324	8	GSE44102	Rhizophagus irregularis	AM inoculated	Affymetrix	Medicago Genome Array	GSM1078949
3	PMID: 24815324	9	GSE44102	Rhizophagus irregularis	AM inoculated	Affymetrix	Medicago Genome Array	GSM1078951
4	PMID: 22034628	10	GSE32208	Rhizophagus irregularis	AM inoculated	Affymetrix	Medicago Genome Array	GSM797965
4	PMID: 22034628	11	GSE32208	Rhizophagus irregularis	AM inoculated	Affymetrix	Medicago Genome Array	GSM797966
4	PMID: 22034628	12	GSE32208	Rhizophagus irregularis	AM inoculated	Affymetrix	Medicago Genome Array	GSM797967
4	PMID: 22034628	13	GSE32208	Glomus mosseae	AM inoculated	Affymetrix	Medicago Genome Array	GSM797968
4	PMID: 22034628	14	GSE32208	Glomus mosseae	AM inoculated	Affymetrix	Medicago Genome Array	GSM797969
4	PMID: 22034628	15	GSE32208	Glomus mosseae	AM inoculated	Affymetrix	Medicago Genome Array	GSM797970
4	PMID: 22034628	16	GSE32208	None	Non-inoculated Control with low P(20 miM)	Affymetrix	Medicago Genome Array	GSM797971
4	PMID: 22034628	17	GSE32208	None	Non-inoculated Control with low P(20 miM)	Affymetrix	Medicago Genome Array	GSM797972
4	PMID: 22034628	18	GSE32208	None	Non-inoculated Control with low P(20 miM)	Affymetrix	Medicago Genome Array	GSM797973

### Reducing heterogenicity between samples of studies: a framework integrating within study rma-normalization, *z*-value transformation, and within samples scaling/quartiling normalization

**Figure 2:**
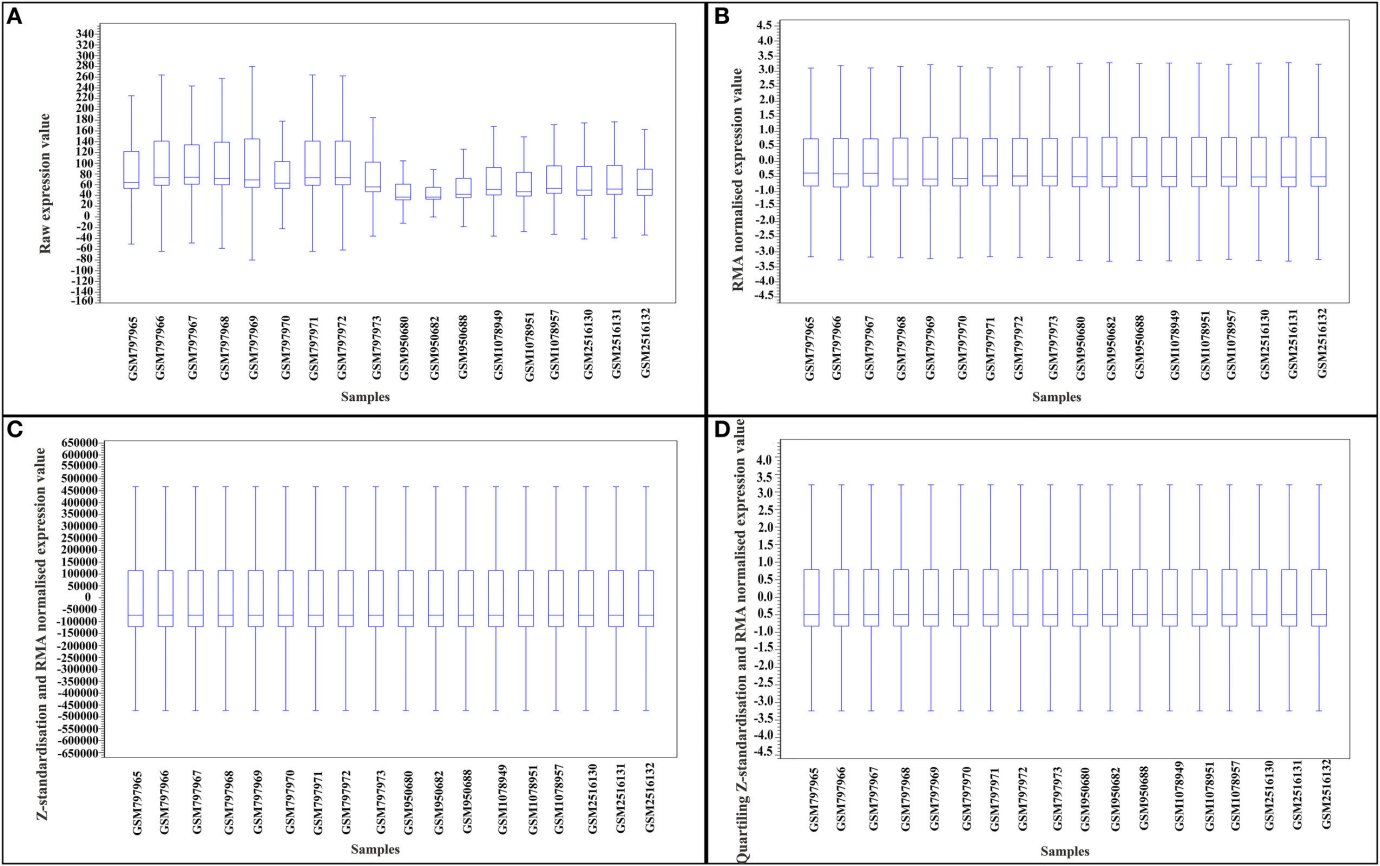
Reducing heterogenicity between samples of studies for direct merging meta-analysis by a three-step normalization process. **(A)** Box plot of raw expression. **(B)** Box plot of expression of RMA-normalized samples. **(C)** Box plot of expression of scaling after Z-standardization and RMA normalized samples. **(D)** Box plot of expression of quartiling after Z-standardization and RMA normalized samples.

Reducing heterogenicity across studies (batch effects) is an essential step for direct combination of expression data in DE meta-analysis. To reduce the batch effects for DM meta-analysis, we developed an integrative approach of within-study RMA-normalization, Z-value transformation, and within samples scaling/quartiling normalization. Figures 2A–D compares the heterogenicity between samples of different studies after different normalization steps. As can be inferred from Figure 2, the proposed framework of normalization and standardization reduced the batch effects and facilitated direct merging of samples from different experiments.

### Meta-genes based biosignature of AM inoculation derived by supervised attribute weighting models

To achieve the transcriptomic signature of AM inoculation, the expression of 33685 genes in inoculated and non-inoculated Medicago roots was mined by 7 feature selection models. Also, the effects of Study (batch effect) and AM type were considered by adding 2 additional polynomial attributes to the expression dataset. The weights of genes as well as Study ID and AM type are presented in Supplementary Table 2. The resulting weights of each feature selection model were normalized into the interval between 0 and 1. The genes announced important by most of the feature selection models with the cut-off > 0.95 were assumed as the key distinguishing genes to form the AM inoculation biosignature.

In total, 681 genes received weight equal or higher than 0.95 by most feature selection algorithms (5 out of 6 models), including UNCERTAINTY, GINI INDEX, CHI SQUARED, RULE, INFO GAIN RATIO, and INFO GAIN. RELIEF was not efficient in gene selection and only gave a high weight to 2 genes out of 33685. Within 681 genes, 180 genes had absolute Z-value difference of 0.5 (> 0.5 or < −0.5) between AM inoculated and non-inoculated (Supplementary Table 3). As presented in Table 2, 73 genes selected by feature selection models were up-regulated with a Z-value difference of > 0.5.

**Table 2:** Transcriptomic biosignature of Arbuscular mycorrhiza (AM) inoculation on Medicago roots derived by integration of supervised attribute weighting models and direct merging meta-analysis.

**Gene**	**Expression statistics of selected meta-gene (up-regulated) in AM inoculated and non-inoculated condition**					**Employed attribute weighting models to discriminate AM inoculation from non-inoculation condition**							**Cut-off**

	**Mean of expression in AM inoculated roots (Z-value standardized)**	**Mean of expression in non-inoculated roots (Z-value standardized)**	***Z*-value difference between inoculated and non-inoculated roots**	**Standard deviation in inoculated condition**	**Standard deviation in non-inoculated condition**	**Weight_Relief**	**Weight_Uncertainty**	**Weight_GiniIndex**	**Weight_Chi Squared**	**Weight_Rule**	**Weight_Info GainRatio**	**Weight_Info Gain**	**Number of models confirmed the importance of gene (Cutoff > 0.95)**
MTR_8g005175	0.03	−0.53	0.56	0.29	0.03	0.02	0.60	1.00	1.00	1.00	1.00	1.00	5
MTR_3g065050	0.85	−0.27	1.12	0.51	0.15	0.01	0.60	1.00	1.00	1.00	1.00	1.00	5
MTR_1g471050	2.15	1.59	0.56	0.17	0.11	0.06	0.75	1.00	1.00	1.00	1.00	1.00	5
MTR_5g092150	1.71	1.09	0.62	0.23	0.07	0.03	0.65	1.00	1.00	1.00	1.00	1.00	5
MTR_7g112963	1.39	0.61	0.78	0.34	0.14	0.01	0.56	1.00	1.00	1.00	1.00	1.00	5
MTR_4g087830	1.64	0.80	0.84	0.20	0.14	0.03	0.64	1.00	1.00	1.00	1.00	1.00	5
MTR_4g113820	1.18	0.35	0.83	0.36	0.17	0.00	0.60	1.00	1.00	1.00	1.00	1.00	5
MTR_6g079630	1.21	−0.09	1.30	0.83	0.06	0.01	0.65	1.00	1.00	1.00	1.00	1.00	5
MTR_4g102400	1.82	0.66	1.15	0.43	0.08	0.01	0.66	1.00	1.00	1.00	1.00	1.00	5
MTR_7g092620	0.93	−0.29	1.22	0.52	0.02	0.01	0.62	1.00	1.00	1.00	1.00	1.00	5
MTR_3g115940	1.50	0.66	0.84	0.18	0.11	0.03	0.65	1.00	1.00	1.00	1.00	1.00	5
MTR_8g006190	0.32	−0.45	0.77	0.40	0.03	0.02	0.67	1.00	1.00	1.00	1.00	1.00	5
MTR_4g129010	1.36	0.63	0.74	0.36	0.11	0.01	0.60	1.00	1.00	1.00	1.00	1.00	5
MTR_7g076960	0.51	−0.64	1.15	0.61	0.17	0.02	0.62	1.00	1.00	1.00	1.00	1.00	5
MTR_7g076920	1.28	0.10	1.19	0.66	0.06	0.00	0.65	1.00	1.00	1.00	1.00	1.00	5
MTR_2g481150	1.06	−0.58	1.64	0.62	0.14	0.01	0.62	1.00	1.00	1.00	1.00	1.00	5
MTR_8g075990	0.24	−0.73	0.96	0.49	0.07	0.02	0.77	1.00	1.00	1.00	1.00	1.00	5
MTR_1g098300	0.68	−0.37	1.05	0.46	0.06	0.01	0.66	1.00	1.00	1.00	1.00	1.00	5
MTR_3g057980	1.38	0.46	0.92	0.34	0.12	0.01	0.66	1.00	1.00	1.00	1.00	1.00	5
MTR_3g034640	1.14	0.31	0.83	0.26	0.09	0.01	0.62	1.00	1.00	1.00	1.00	1.00	5
MTR_7g080180	1.72	1.13	0.59	0.25	0.10	0.04	0.56	1.00	1.00	1.00	1.00	1.00	5
MTR_8g074920	1.20	0.70	0.50	0.25	0.11	0.02	0.56	1.00	1.00	1.00	1.00	1.00	5
MTR_3g064090	0.50	−0.14	0.64	0.21	0.10	0.00	0.73	1.00	1.00	1.00	1.00	1.00	5
MTR_6g015020	1.56	0.97	0.59	0.20	0.06	0.04	0.64	1.00	1.00	1.00	1.00	1.00	5
MTR_0088s0100	0.26	−0.70	0.96	0.54	0.09	0.02	0.64	1.00	1.00	1.00	1.00	1.00	5
MTR_1g115230	0.78	0.28	0.50	0.30	0.03	0.01	0.62	1.00	1.00	1.00	1.00	1.00	5
MTR_5g018610	1.85	0.46	1.38	0.59	0.08	0.01	0.63	1.00	1.00	1.00	1.00	1.00	5
MTR_3g079620	1.52	−0.70	2.21	0.88	0.25	0.01	0.69	1.00	1.00	1.00	1.00	1.00	5
MTR_3g045440	1.05	−0.65	1.71	0.49	0.10	0.00	0.65	1.00	1.00	1.00	1.00	1.00	5
MTR_5g019460	1.69	1.17	0.51	0.15	0.03	0.07	0.62	1.00	1.00	1.00	1.00	1.00	5
MTR_2g088700	1.21	0.66	0.56	0.22	0.06	0.02	0.68	1.00	1.00	1.00	1.00	1.00	5
MTR_1g069725	1.11	0.13	0.97	0.38	0.07	0.00	0.60	1.00	1.00	1.00	1.00	1.00	5
MTR_1g103090	−0.03	−0.78	0.76	0.65	0.06	0.02	0.79	1.00	1.00	1.00	1.00	1.00	5
MTR_8g075330	0.78	−0.22	1.00	0.46	0.08	0.01	0.62	1.00	1.00	1.00	1.00	1.00	5
MTR_3g078730	−0.21	−0.76	0.56	0.45	0.04	0.02	0.76	1.00	1.00	1.00	1.00	1.00	5
MTR_5g094210	0.07	−0.84	0.91	0.63	0.07	0.03	0.67	1.00	1.00	1.00	1.00	1.00	5
MTR_3g112460	0.34	−0.52	0.86	0.46	0.08	0.02	0.62	1.00	1.00	1.00	1.00	1.00	5
MTR_5g045470	2.57	0.81	1.76	0.36	0.34	0.02	0.70	1.00	1.00	1.00	1.00	1.00	5
MTR_6g043700	0.86	−0.56	1.42	0.82	0.21	0.02	0.60	1.00	1.00	1.00	1.00	1.00	5
MTR_5g031160	1.65	−0.56	2.20	0.94	0.08	0.00	0.73	1.00	1.00	1.00	1.00	1.00	5
MTR_1g115195	1.22	0.26	0.96	0.37	0.28	0.00	0.60	1.00	1.00	1.00	1.00	1.00	5
MTR_8g087710	1.85	0.65	1.20	0.56	0.08	0.01	0.66	1.00	1.00	1.00	1.00	1.00	5
MTR_4g075690	1.53	0.73	0.80	0.49	0.16	0.01	0.65	1.00	1.00	1.00	1.00	1.00	5
MTR_2g461970	1.94	1.12	0.82	0.20	0.07	0.04	0.63	1.00	1.00	1.00	1.00	1.00	5
MTR_8g036050	1.45	0.16	1.29	0.46	0.06	0.01	0.65	1.00	1.00	1.00	1.00	1.00	5
MTR_2g010580	1.52	0.84	0.68	0.25	0.02	0.03	0.65	1.00	1.00	1.00	1.00	1.00	5
MTR_8g072010	1.55	1.00	0.55	0.16	0.22	0.03	0.71	1.00	1.00	1.00	1.00	1.00	5
MTR_4g069810	1.68	0.30	1.38	0.41	0.17	0.01	0.60	1.00	1.00	1.00	1.00	1.00	5
MTR_8g044230	0.66	0.08	0.58	0.36	0.06	0.01	0.59	1.00	1.00	1.00	1.00	1.00	5
MTR_7g105560	1.71	1.02	0.69	0.17	0.05	0.05	0.72	1.00	1.00	1.00	1.00	1.00	5
MTR_6g027840	1.90	0.84	1.06	0.32	0.13	0.02	0.59	1.00	1.00	1.00	1.00	1.00	5
MTR_6g006990	0.73	−0.56	1.29	0.50	0.07	0.01	0.60	1.00	1.00	1.00	1.00	1.00	5
MTR_8g068265	1.79	1.22	0.57	0.16	0.10	0.05	0.66	1.00	1.00	1.00	1.00	1.00	5
MTR_6g029180	1.58	0.82	0.77	0.19	0.08	0.04	0.62	1.00	1.00	1.00	1.00	1.00	5
MTR_4g081190	1.01	−0.50	1.51	0.54	0.10	0.01	0.68	1.00	1.00	1.00	1.00	1.00	5
MTR_7g082660	0.13	−0.52	0.66	0.21	0.19	0.02	0.70	1.00	1.00	1.00	1.00	1.00	5
MTR_5g075400	1.26	0.04	1.22	0.62	0.10	0.00	0.68	1.00	1.00	1.00	1.00	1.00	5
MTR_3g083630	2.04	1.23	0.82	0.27	0.17	0.03	0.59	1.00	1.00	1.00	1.00	1.00	5
MTR_8g022270	1.73	−0.12	1.85	0.67	0.09	0.00	0.68	1.00	1.00	1.00	1.00	1.00	5
MTR_8g018650	2.42	1.33	1.09	0.32	0.11	0.03	0.60	1.00	1.00	1.00	1.00	1.00	5
MTR_7g098230	0.81	−0.33	1.14	0.28	0.03	0.00	0.66	1.00	1.00	1.00	1.00	1.00	5
MTR_2g089100	0.70	−0.10	0.80	0.26	0.15	0.00	0.61	1.00	1.00	1.00	1.00	1.00	5
MTR_3g057970	1.06	0.45	0.61	0.26	0.05	0.01	0.64	1.00	1.00	1.00	1.00	1.00	5
MTR_3g058000	0.40	−0.45	0.85	0.38	0.13	0.01	0.66	1.00	1.00	1.00	1.00	1.00	5
MTR_1g050550	0.57	−0.22	0.79	0.20	0.06	0.00	0.65	1.00	1.00	1.00	1.00	1.00	5
MTR_7g077110	1.26	−0.67	1.93	0.47	0.20	0.00	0.63	1.00	1.00	1.00	1.00	1.00	5
MTR_7g077050	0.96	−0.97	1.93	0.47	0.07	0.00	0.65	1.00	1.00	1.00	1.00	1.00	5
MTR_2g068950	1.07	0.36	0.71	0.26	0.05	0.01	0.63	1.00	1.00	1.00	1.00	1.00	5
MTR_4g076490	1.09	0.30	0.79	0.16	0.06	0.02	0.65	1.00	1.00	1.00	1.00	1.00	5
MTR_6g005630	0.49	−0.47	0.96	0.38	0.07	0.01	0.62	1.00	1.00	1.00	1.00	1.00	5
MTR_1g094130	1.82	1.30	0.52	0.26	0.18	0.03	0.58	1.00	1.00	1.00	1.00	1.00	5
MTR_8g068050	0.99	0.36	0.63	0.25	0.04	0.01	0.59	1.00	1.00	1.00	1.00	1.00	5
MTR_5g026730	0.50	−0.57	1.07	0.42	0.04	0.01	0.66	1.00	1.00	1.00	1.00	1.00	5
Study ID						0.03	0.24	0.17	0.18	0.00	0.05	0.26	0
Type of AM						0.00	0.90	1.00	1.00	1.00	0.32	1.00	4

*The role of the following 73 up-regulated meta-genes (derived by direct merging meta-analysis of different experiments) was re-confirmed by feature selection (attribute weighting) models. To this end, seven feature selection models including RELIEF, UNCERTAINTY, GINI INDEX, CHI SQUARED, RULE, INFO GAIN, and INFO GAIN RATIO evaluated the relevance of 33685 genes as well as Study ID and AM type in discriminating AM inoculated roots from non-inoculated ones. The resulting weights of each feature selection model were normalized into the interval between 0 and 1. The upregulated genes in response to AM inoculation with a Z-value difference of >0.5, announced important by most of feature selection models, intersection of weighting methods with various statistical backgrounds (cutoff > 0.95), were selected as the key distinguishing genes to form the AM inoculation biosignature*.

The 73 highly upregulated genes responding to AM inoculation, as transcriptomic biosignature (Table 2), contain important classes of genes including AP2 domain class transcription factors (MTR_6g029180), GRAS family transcription factors (MTR_1g069725 and MTR_2g089100), cyclin-dependent kinase (MTR_1g098300), receptors [lectin receptor kinase (MTR_8g068050), LRR receptor-like kinase (MTR_8g044230), cysteine-rich RLK (MTR_3g064090), and LRR receptor-like kinase (MTR_8g044230)], trypsin inhibitor (MTR_5g045470), Nodule Cysteine-Rich secreted peptide (MTR_3g065050), early nodulin 93 (MTR_4g113820), and transporters (MTR_4g081190, MTR_4g081190, MTR_8g022270, MTR_8g087710, and MTR_1g050550) (Table 3).

**Table 3:** Description of highly upregulated genes in transcriptomic signature of Arbuscular mycorrhiza (AM) inoculation on Medicago roots.

**Class of protein**	**Subclass**	**Member from upregulated transcriptomic signature responding to AM inoculation**
Transcription factor	GRAS family transcription factor	*MTR_1g069725, MTR_2g089100*
	AP2 domain class transcription factor	*MTR_6g029180*
	Zinc finger, C3HC4 type (RING finger) protein	*MTR_5g026730*
Phosphate synthase	1-deoxy-D-xylulose-5-phosphate synthase	*MTR_8g068265*
	Geranylgeranyl pyrophosphate synthase	*MTR_5g019460*
Transporters	Phospholipase A1 transporter	*MTR_4g087830*
	ABC transporter B family protein	*MTR_4g081190, MTR_8g022270*
	Major intrinsic protein (MIP) family transporter	*MTR_8g087710*
	MFS transporter	*MTR_1g050550*
	Peptide transporter	*MTR_3g112460, MTR_7g098230*
Cyclin-dependent kinase	Cyclin-dependent kinase	*MTR_1g098300*
Receptors	Cysteine-rich RLK (receptor-like kinase) protein	*MTR_3g064090*
	Lectin receptor kinase	*MTR_8g068050*
	LRR receptor-like kinase	*MTR_8g044230*
Nodule proteins	Nodule Cysteine-Rich (NCR) secreted peptide	*MTR_3g065050*
	Early nodulin 93	*MTR_4g113820*
Tyrosine kinase	Tyrosine kinase family protein	*MTR_4g129010*
Cytochrome	Cytochrome P450	*MTR_3g057970, MTR_3g057980, MTR_3g058000, MTR_5g092150, MTR_7g092620*
Oxidase	L-ascorbate oxidase	*MTR_3g078730, MTR_3g078730*
	Multi-copper oxidase-like protein	*MTR_4g075690*
Serine carboxypeptidase	Serine carboxypeptidase-like protein	*MTR_3g079620, MTR_7g080180*
	Biotin carboxyl carrier acetyl-CoA carboxylase	*MTR_6g015020*
Inhibitor	Inhibitor of trypsin and hageman factor-like protein	*MTR_5g045470*
legume specific proteins	Legume lectin beta domain protein	*MTR_5g031160*
Cysteine-rich protein	CAP, cysteine-rich secretory protein, antigen 5	*MTR_2g010580*
Tetrahydrodipicolinate synthase	4-hydroxy-tetrahydrodipicolinate synthase	*MTR_8g036050*
Hydrolase	Glycoside hydrolase	*MTR_8g075330, MTR_8g075990*
	Epoxide hydrolase	*MTR_7g112963*
Chitinase	Chitinase	*MTR_6g079630*
Alginate lyase	Alginate lyase	*MTR_6g043700*
Oxidoreductase	2OG-Fe(II) oxygenase family oxidoreductase	*MTR_2g068950*
Glucan-protein synthase	Alpha-1,4-glucan-protein synthase protein	*MTR_2g461970*
Arginase	Arginase family protein	*MTR_0088s0100*
Beta-carotene isomerase	Beta-carotene isomerase D27	*MTR_1g471050*
Carbonic anhydrase	Carbonic anhydrase family protein	*MTR_6g006990*
Glucosidase	Glucan endo-1,3-beta-glucosidase	*MTR_4g076490*
Glutathione S-transferase	Glutathione S-transferase	*MTR_1g115195*
Oxygen enhancer protein	Oxygen-evolving enhancer protein	*MTR_8g005175*
Pectinacetylesterase	Pectinacetylesterase family protein	*MTR_8g072010*
Polygalacturonase	Polygalacturonase	*MTR_6g005630*
Prolyl oligopeptidase	Prolyl oligopeptidase family protein	*MTR_1g115230*
Lipoxygenase	Seed linoleate 9S-lipoxygenase	*MTR_8g018650*
Transmembrane	Seven transmembrane MLO family protein	*MTR_3g115940*
Squalene synthase	Squalene/phytoene synthase	*MTR_3g083630*
Proteolysis	Subtilisin-like serine protease	*MTR_4g102400*
Syntaxin	Syntaxin of plants 122 protein	*MTR_2g088700*

*The 73 up-regulated genes responding to AM inoculation were derived by meta-analysis and supervised machine learning analysis*.

**Figure 3:**
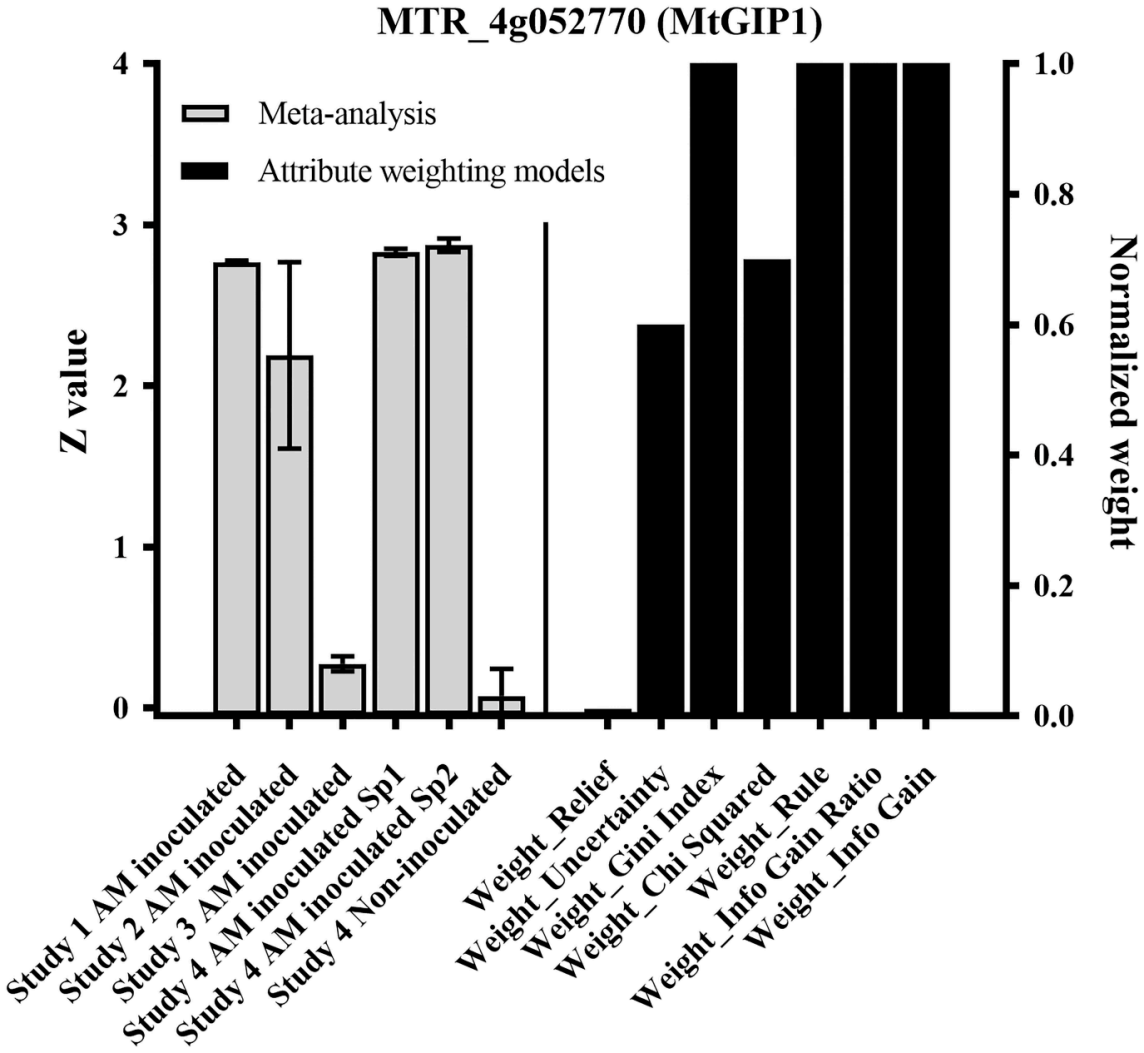
MtGIP1 received high weights (values) in 6 out of 7 of the employed attribute weighting models in order to distinguish Arbuscular mycorrhiza (AM)-inoculated samples from non-inoculated ones, according to UNCERTAINTY, GINI INDEX, Chi Squared, RULE, INFO GAIN RATIO, and INFO GAIN models, where weighting closer to 1 shows a higher relevance (importance) of gene according to the respected model. The normalized expression value of MtGIP1 in AM-inoculated and non-inoculated samples are also presented.

Within the previously-reported AM markers (Hogekamp et al., 2011), MtGIP1 received high weights (values) in 6 out of 7 of the employed attribute weighting models in order to distinguish AM-inoculated from non-inoculated samples. Figure 3 visualizes the high weights assigned to MtGIP1 by UNCERTAINTY, GINI INDEX, CHI SQUARED, RULE, INFO GAIN RATIO, and INFO GAIN models where weighting closer to 1 shows a higher relevance (importance) of gene according to the respective model. Also, Figure 3 presents the normalized expression value of MtGIP1 in AM-inoculated and non-inoculated samples. MtGIP1 can be assumed to be a reliable AM colonization marker as its predictive powers is confirmed by previous individual studies as well as the combined meta-analysis performed here.

### Supervised machine learning models showed that the batch effect (heterogenicity between experiments) is remarkably reduced

The results of attribute weighting (feature selection) models presented in Table 2 show that the effect of Study ID (batch effect) is not significant in deriving the signature of AM inoculation. Interestingly, while 180 genes were selected by most of attribute weighting models to discriminate AM-inoculated from non-inoculated roots (Supplementary Table 3, Table 2), none of the models selected Study ID.

**Figure 4:**
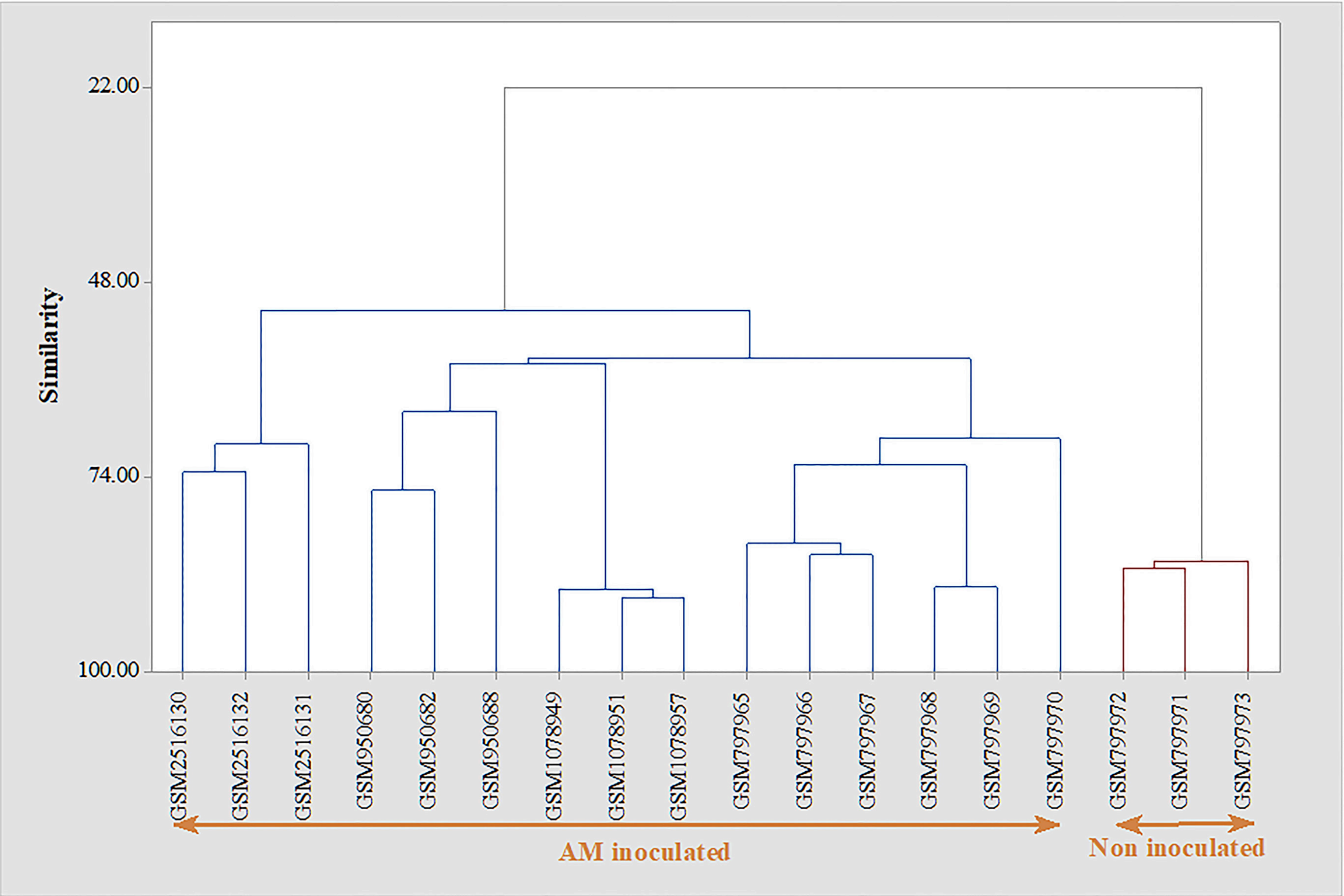
Evaluation of the developed transcriptomic signature of Arbuscular mycorrhiza (AM) inoculation in discrimination of inoculated from non-inoculated samples. Clustering was performed based on Average Linkage method and Euclidean distance. Clustering based on the identified transcriptomic signature was able to efficiently distinguish between AM inoculated from non-inoculated samples.

In line with this finding, clustering analysis (Figure 4) showed that the developed AM inoculation signature is able to discriminate between AM-inoculated and non-inoculated samples. As presented in Figure 4, while the AM-inoculated samples had more than 50% similarity to transcriptomic signature genes, this similarity decreased to 22% with AM non-inoculated genes.

### The transcriptomic signature of am inoculation identifies the involvement of hydrolase activity, phosphorylation, cell wall organization, and transport, based on computational systems biology analysis

**Figure 5:**
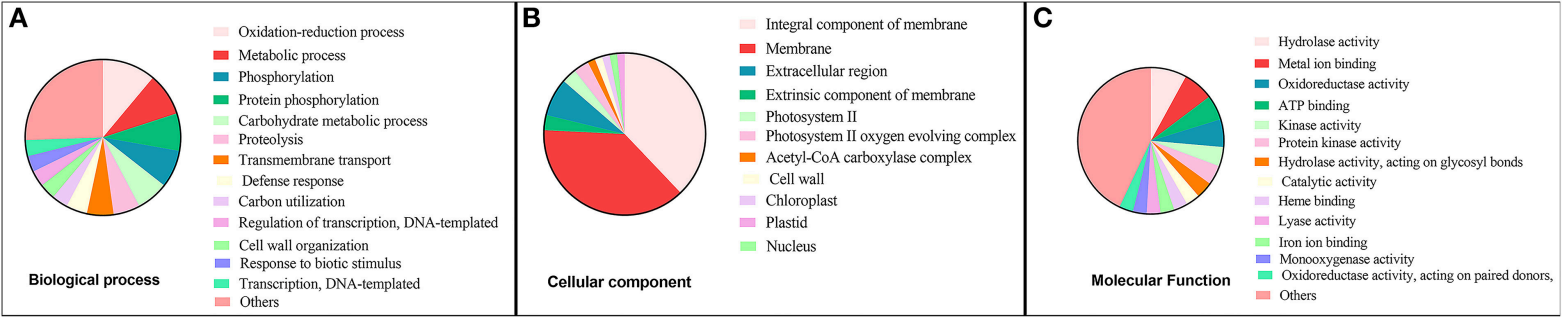
Gene Ontology (GO) distribution of upregulated genes in transcriptomic signature of successful Arbuscular mycorrhiza (AM) colonization in roots of *Medicago truncatula*. **(A)** GO at Biological process level, **(B)** GO at Cellular component level, **(C)** GO at Molecular function level.

Functional annotation of the transcriptomic signature based on GO analysis showed that a majority of genes in the transcriptomic signature encode membrane proteins and are involved in cell wall organization, membrane transport, proteolysis, and oxidoreductase activities (Supplementary Table 4). GO distribution of the transcriptomic signature is presented in Figure 5.

The isoprenoid biosynthetic/metabolic process and the lipid biosynthetic/metabolic process were statistically significant (enriched) biological processess that can be activated by upregulated genes of transcriptomic signature (*p*-value FDR < 0.05) (Figure 5A). Response to stimulus was another interesting aspect enriched in the GO. In the cellular component GO category, genes involved in response to AM colonization, including cell wall and external encapsulating structure, showed high enrichment (Figure 5B). In terms of Molecular Function, transferase and hydrolase activities were significantly enriched (Figure 5C) (Supplementary Table 4). In line with this finding, analysis of transcriptome response of *Medicago truncatula* to *Glomus mosseae* and *Rhizophagus irregularis* by Hohnjec et al. (2005), showed that 201 plant genes were significantly co-induced at least 2-fold. These genes were related to functions such as nitrate, ion, and sugar transporter, and enzymes involved in secondary metabolism, proteases, and Kunitz-type protease inhibitors.

### Overrepresented transcription factor binding sites on promoter regions of upregulated AM colonization transcriptomic signature enabled discovery of potential master regulators of AM colonization

Transcription factors with enriched binding sites on promoter regions of the transcriptomic signature are candidates for “common master regulators” (Hosseinpour et al., 2013; Mahdi et al., 2014; Alanazi and Ebrahimie, 2016; Alanazi et al., 2018). Transcription factor matrix families with statistically enriched (*p* < 0.01) binding sites in the highly upregulated meta-gene transcriptomic signature (top 20 genes) of successful AM inoculation is presented in Table 4 and Supplementary Table 6. The common enriched TFs were: P$FLO2, P$SEF3, P$TERE, P$ASRC, P$CARM, P$TOEF, P$SEF4, P$LREM, P$MYBL, P$CAAT, P$GTBX, and P$WOXF (Table 4 and Supplementary Table 6).

**Table 4:** Transcription factors matrix families with frequent binding sites on promoter regions of the top 20 upregulated genes during successful Arbuscular mycorrhiza (AM) colonization as potential master regulators of AM colonization.

**TF Matrix Family**	**TF Family Description**	**Example of TFs**	**Binding domain of TF**	***p*-value**	**NO binding sites**	**NO gens with TF**	**Top 20 upregulated genes in AM inoculation signature**																			
							**MTR_3g045440**	**MTR_2g068950**	**MTR_2g481150**	**MTR_8g022270**	**MTR_7g077110**	**MTR_5g018610**	**MTR_7g092620**	**MTR_4g081190**	**MTR_1g069725**	**MTR_5g045470**	**MTR_4g069810**	**MTR_8g036050**	**MTR_8g068050**	**MTR_6g043700**	**MTR_3g079620**	**MTR_7g077050**	**MTR_6g079630**	**MTR_5g031160**	**MTR_6g006990**	**MTR_5g094210**
P$FLO2	Floral homeotic protein APETALA 2	AP2	AP2 domain	2.05E-05	71	20	2	4	3	7	3	1	3	5	7	3	4	4	2	2	2	2	1	2	12	2
P$SEF3	Soybean embryo factor 3	SEF3	not specified	0.000271	21	14	4	2	1	1	1	0	2	1	1	0	0	2	1	0	1	0	1	2	0	1
P$TERE	Tracheary-element-regulating cis-elements, conferring TE-specific expression	TERE		0.000345	42	18	0	4	4	5	2	1	1	2	3	1	4	3	1	1	2	2	2	1	3	0
P$ASRC	AS1/AS2 repressor complex	AS1, AS2	not specified	0.000432	103	20	5	10	3	11	3	7	9	3	4	1	5	8	3	2	9	3	4	1	11	1
P$CARM	CA-rich motif	CARM	not characterized	0.000613	42	17	3	2	0	5	1	0	2	6	1	0	2	1	4	1	1	1	2	1	7	2
P$TOEF	Target of early activation tagged factors	RAP2.7, TOE2	AP2 domain	0.001698	56	20	3	5	1	5	2	3	3	2	4	2	5	2	2	1	2	2	6	2	3	1
P$SEF4	Soybean embryo factor 4	SEF4	not specified	0.002042	24	13	1	4	1	3	0	1	0	3	1	1	2	0	1	0	2	0	0	3	1	0
P$MYBL	MYB-like proteins	MYB, AS1, AS2, FIF1		0.002825	293	20	13	20	6	53	6	10	21	28	10	6	12	10	15	2	20	7	4	18	26	6
P$CAAT	CCAAT binding factors	LEC1, NF-YA1,NF-YB1	heterotrimeric transcription factor	0.003168	92	19	3	11	0	17	3	3	4	10	6	6	2	4	2	3	5	1	3	1	7	1
P$GTBX	GT-box elements	ASIL1, S1FA, GT2, GT1		0.006813	454	20	22	60	10	61	7	16	26	27	24	14	26	12	22	8	22	7	11	16	53	10
P$WOXF	WUS homeobox-containing protein family	WOX13	homeodomain	0.009397	73	18	4	6	1	9	2	1	6	7	5	0	4	4	3	2	4	5	3	2	5	0

**Figure 6:**
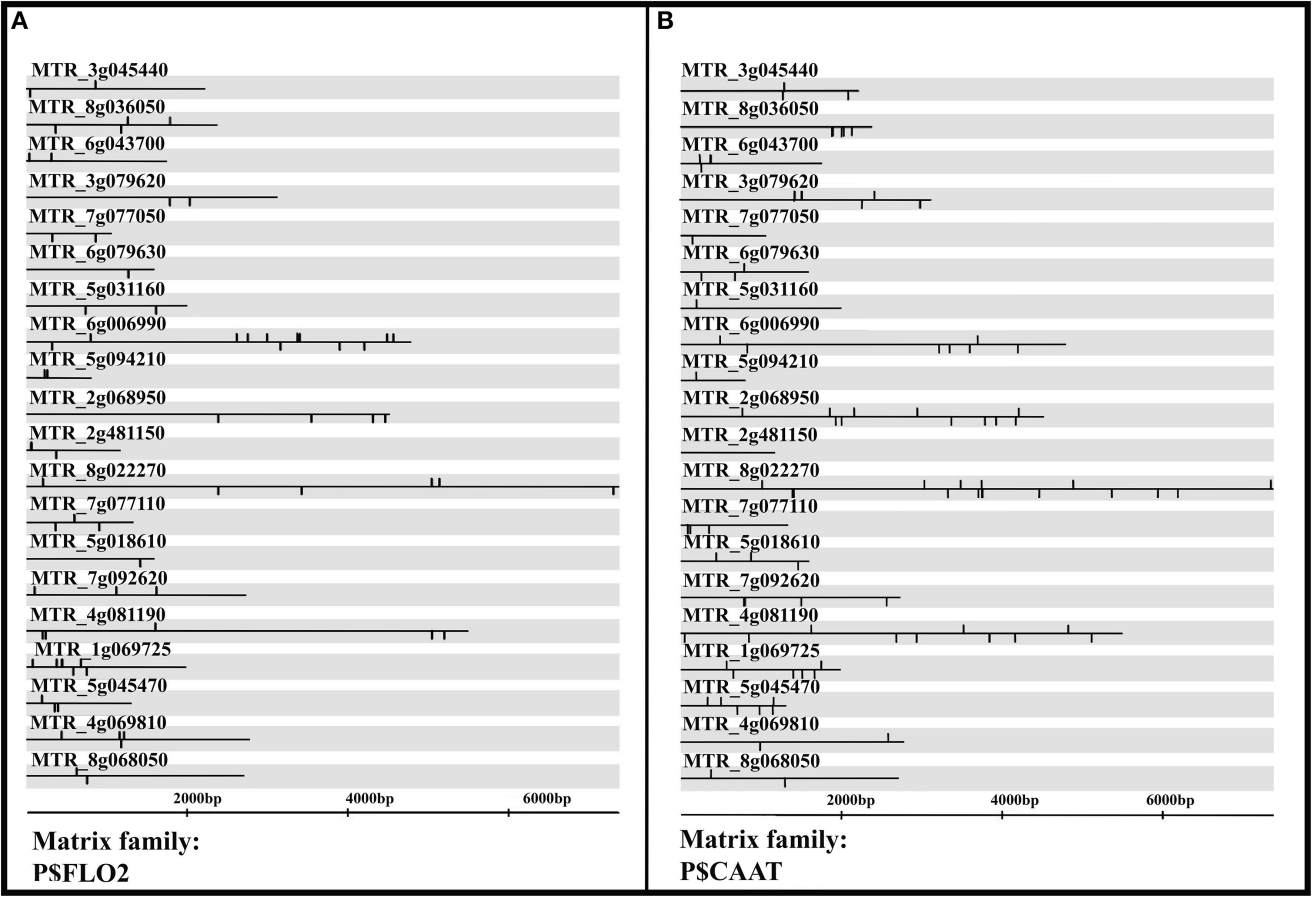
P$CAAT **(A)** and P$FLO2 **(B)** transcription factor matrix families were master regulators of successful Arbuscular mycorrhiza (AM) colonization in roots of *Medicago truncatula* with enriched (high number of) binding sites on promoter regions of the top 20 upregulated genes during successful AM colonization. P$FLO2 transcription factor matrix family contains transcription factors with AP2 domain structure and ethylene-responsive element (ERE) binding. P$CAAT matrix family includes CCAAT binding transcription factors, such as NF-YA, NF-YB, and LEC1.

Promoter analysis of upregulated genes in the AM colonization signature identified the P$FLO2 matrix family as one of the master regulators due to the highest number of binding sites (71) within promoter regions of all the 20 highly upregulated genes (0.0000204538). The P$FLO2 family contains transcription factors with AP2 domains and ethylene-responsive element (ERE) binding. (Table 4, Figure 6A, and Supplementary Table 6).

The P$CAAT matrix family that includes CCAAT binding transcription factors, such as NF-YA, NF-YB, and LEC1, was selected as another common TF (master regulator) with a low *p*-value (*p* = 0.00316799) in common TF analysis. P$CAAT matrix had binding sites on promoter regions in 19 out of the 20 upregulated genes in the AM inoculation signature with the total number of 92 binding sites (Table 4, Supplementary Table 6, and Figure 6B).

P$SEF3 (soybean embryo factor 3) and P$SEF4 (soybean embryo factor 4), that contain SEF3 and SEF4 transcription factors, had a significantly (p- value < 0.01) high number of interactions with the top 20 upregulated genes in the AM colonization signature and these were tentatively identified as potential key regulators of AM colonization (Table 4, Supplementary Table 6). The P$TERE matrix family that confers transcription factor-specific expression was also enriched in promoter regions of upregulated genes after AM colonization.

Another enriched transcription factor matrix family was P$TOEF that contains the AP2 domain in its structure and is involved in early activation/response (Table 4, Supplementary Table 6). RAP2.7 and TOE2 are well-known members of this matrix family. GO analysis showed that this matrix family is involved in organ morphogenesis.

A matrix family involved in response to fungal colonization, P$ASRC, had 103 binding sites on promoter regions of the top 20 upregulated genes in AM successful colonization with *p*-value of 0.000432195. AS1, AS2 are members of this transcription factor matrix family.

The transcription factor family of MYB-like proteins, belonging to the P$MYBL matrix family, was also enriched (total of 293 binding sites and *p*-value of 0.002825). This family includes important transcription factors, including *MYB, AS1, AS2*, and *FIF1*.

### The AM colonization meta-signature showed high repeatability in an independent rna-seq experiment of AM colonization

**Figure 7:**
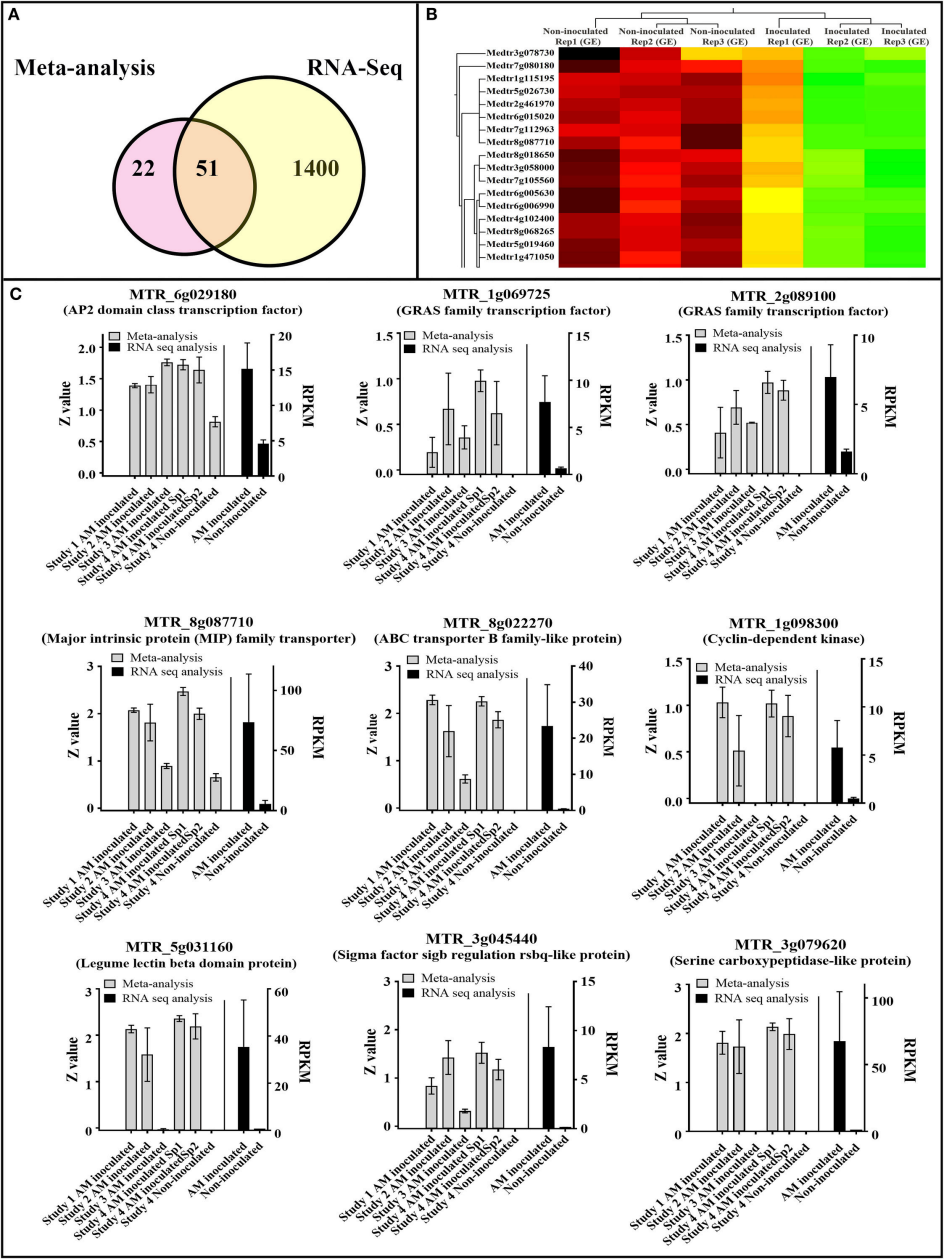
High correspondence between RNA-seq data of Arbuscular mycorrhiza (AM) colonization and the identified AM colonization meta-signature in this study, derived from integration of meta-analysis with supervised attribute weighting models. **(A)** 51 out of 73 (70%) of the upregulated genes in the developed transcriptomic biosignature ofcolonization were also upregulated in the RNA-seq data of AM colonization with FDR-corrected *p* < 0.01. **(B)** The identified AM colonization meta-signature was able to accurately discriminate AM-inoculated samples from non-inoculated ones. **(C)** Visualization of the expression of some important genes of AM colonization signature in original experiments (based on standardized *Z*-value of expression) and RNA-seq experiment [based on RPKM (Reads Per Kilobase of transcript per Million mapped reads)].

In an independent RNA-seq experiment, we observed high correspondence between RNA-seq data of AM colonization and the identified AM colonization signature in this study, derived from integration of meta-analysis with supervised attribute weighting models (Figure 7, Supplementary Table 7). Fifty-one of 73 (70%) of the upregulated genes in the developed transcriptomic biosignature of AM colonization were also upregulated in the independent RNA-seq data of AM colonization with FDR-corrected *p* < 0.01 (Figure 7A). Noticeably, the identified AM colonization meta-signature was able to discriminate accurately between AM-inoculated samples and non-inoculated ones (Figure 7B). High correspondence between the expression of some of the important genes of the AM-colonization signature in the original microarray experiments (based on standardized *Z*-value of expression) and the expression of those genes in the RNA-seq experiment [based on RPKM (Reads Per Kilobase of transcript per Million mapped reads) are visualized in Figure 7C, including the AP2 domain class transcription factor (*MTR_6g029180*), members of GRAS family of transcription factor (*MTR_1g069725, MTR_2g089100*), Cyclin-dependent kinase (*MTR_1g098300*), MIP family transporter (*MTR_8g087710*), ABC transporter B family-like protein (*MTR_8g022270*), Legume lectin beta domain protein (*MTR_5g031160*), Sigma factor sigb regulation rsbq-like protein (*MTR_3g045440*), and Serine carboxypeptidase-like protein (*MTR_3g079620*).

In short, after quality control and trimming, 18772504, 19678186, 22009349, 18982223, and 19364133 remained in 3 AM-inoculated and non-inoculated samples. High efficiency of mapping to genes (more than 95%) was observed in all samples. Supplementary Table 8 presents RNA-seq based differential expression analysis of *Medicago truncatula* response to AM inoculation compared to non-AM inoculation.

## Discussion

Finding a biosignature/predictors based on a single transcriptomic experiment is a major challenge due to a large prediction error caused by a large number of independent predictors (genes) and a restricted number of observations (replications) (Baseri et al., 2011). Also of concern is the repeatability of a selected subset of a gene derived from a single experiment/condition. Inter-species analysis of a range of experiments by meta-analysis and machine learning techniques is able to deal with theseshortcomings, leading to the generation of a robust and repeatable biosignature (Farhadian et al., 2018b). Meta-analysis has received increased attention in recent years because of its remarkable potential to increase the statistical power and generalizability of single study analysis (Farhadian et al., 2018a; Sharifi et al., 2018). Meta-analysis not only reinforces the findings of the individual studies, but is also may identify new undetected outcomes/patterns in single studies as meta-analysis considers the direction/trend of variables in each experiment to find the consistent, robust and repeatable patterns in all experiments (Sharifi et al., 2018). The inter-species DE-based meta-analysis employed in this study had more samples and stronger statistical power and was successful in achieving a statistically-reliable transcriptomic biosignature of successful AM inoculation, independent from the study. In addition, the biosignature was repeatable and discriminative when a new and independent RNA-seq experiment was used for its validation. Due to the availability of *Medicago truncatula* transcriptomic data (as a model plant), the meta-analysis was solely performed on this plant resulting in the identification of a robust and high performance transcriptomic signature of AM colonization. However, in non-model plants with the subsequent generation of new transcriptomic data, it will be necessary the identified *Medicago truncatula*-derived transcriptomic signature of AM colonization will need further examination.

In DE-based meta-analysis, it is crucial to adjust for batch effects before combining expression datasets. Heterogenicity (batch effects) is the major concern in meta-analysis of expression data (Leek and Storey, 2007; Ramasamy et al., 2008). In this study, we developed a new approach for reducing batch effects and direct merging meta-analysis by combination of meta-analysis, multi-step normalization, and supervised attribute weighting models. We observed that quartiling outperforms the scaling approach in reducing the batch effect. Heterogenicity-reducing based on the quartiling approach has been used extensively for knowledge discovery and pattern recognition in large data analysis, particularly in integrated classification and association-rule mining (CBA) algorithm (Kargarfard et al., 2015, 2016). As an example, CBA analysis of quartiled protein and DNA measurements was able to find a biosignature for increased host range and the emergence of an outbreak in influenza (Kargarfard et al., 2016). Supervised machine learning has brought new possibilities to predictive studies (Bakhtiarizadeh et al., 2014a; Ebrahimi et al., 2014; Zinati et al., 2014; Ebrahimie et al., 2018b). Supervised attribute weighting (feature selection) algorithms are techniques for reducing the variables and identifying a subset of highly relevant ones in order to improve the efficiency of classification algorithms (Rosario and Thangadurai, 2015). The capability to simultaneously analyse both categorical and numerical features, power to analyse large data, and the ability to produce various predictive algorithms with diverse statistical backgrounds are distinguished features of supervised machine learning models (Ebrahimie et al., 2011; Shekoofa et al., 2014). The possibility to include the categorical variables in predictive models can remarkably decrease the heterogenicity across studies as the batch effects and other non-biological experimental variation were incorporated in the models (Shekoofa et al., 2014). In this study, different experiments or types of AM were added as variables and analyse in the predictive model that resulted in remarkable control of batch effect. This possibility is very limited in traditional multivariate or regression models.

The identified meta-genes of successful AM colonization, derived by integration of meta-analysis with supervised attribute weighting models, was able to discriminate efficiently between AM-inoculated and non-inoculated samples. As a validation analysis, the developed signature showed high performance in distinguishing AM-colonized roots from non-inoculated ones in an independent RNA-seq experiment. Recently, integration of supervised machine learning algorithms with meta-analysis has been used to identify a mastitis bio-signature and early prediction of its occurrence (Ebrahimie et al., 2018a; Sharifi et al., 2018). The developed integrative approach in this study, comprising multi-step normalization, direct-merging meta-analysis, and supervised attribute weighting models, is platform-independent approach. By subsequent generation of more RNA-seq data, the developed pipeline may be employed for biosignature discovery in RNA-seq transcriptomic data, integration of microarray and transcriptomic data as is possible using some other NGS platforms, such as ChIP-Seq and SNP to perform meta-analysis on significant peaks in ChIP-Seq experiments and frequency of SNPs in genome-wide experiments.

The core 73 upregulated genes in the developed transcriptomic biosignature contain novel regulators of AM colonization including two transcription factors from the GRAS family (MTR_1g069725, MTR_2g089100), one transcription factor from AP2 domain class (MTR_6g029180), and one Zinc finger protein. It has been documented that the GRAS-type transcription factors, such as NSP1 (Nodulation Signaling Pathway1) and NSP2, play essential signaling functions in promoting both Rhizobium nodulation and mycorrhizal colonization (Kaló et al., 2005; Smit et al., 2005; Liu et al., 2011; Gobbato et al., 2012). Another transcription discovered factor, MTR_6g029180, has an AP2 domain in this structure. Interestingly, it has been reported that ERF transcription factors with a highly conserved AP2 DNA-binding domain are necessary for nodulation and symbiosis (Middleton et al., 2007). Cyclin-dependent kinase (MTR_1g098300) was another highly upregulated gene in the signature of successful AM colonization in this study. Mycorrhizal colonization is classified as postembryonic development of plant organs that need a constant interplay between the cell cycle and developmental programs (Kondorosi and Kondorosi, 2004). Cyclin-dependent kinase controls cell cycle and plays the key role in endoreduplication and activation of the anaphase-promoting complex during symbiotic cell development (Kondorosi and Kondorosi, 2004). The discovery of the essential transcription factors of successful mycorrhizal colonization and symbiosis in the developed biosignature highlights the robustness and applicability of meta-analysis in the AM colonization signature discovery and the importance of the developed transcriptomic signature. The biosignature obtained here provides a platform for increasing the efficiency of AM inoculation in future by finding accelerator AM colonization agents, such as small molecules/chemicals, and manipulating the expression of key genes in the biosignature.

The reasons that some previously-reported AM-associated genes were not identified in the AM meta-signature might be: (1) there are other genes with higher and more repeatable expression in response to AM induction and colonization which are, as a result, selected. These new candidates have higher preference over some of the previously-known biomarkers of AM symbiosis, (2) some AM markers might interact with the type of AM and consequently these will not appear in cross-species meta-analysis, and (3) some AM markers may interact with a specific condition or timing of AM symbiosis. As example, mycorrhiza-specific phosphate transporter seems to be more closely related to P homeostasis rather than colonization as the phosphate transporter mediates early root responses to phosphate status in non-mycorrhizal roots (Volpe et al., 2016).

Reinforcing the importance of the existence of AP2 transcription factors in the upregulated transcriptomic signature of AM colonization, promoter analysis demonstrated that the P$FLO2 transcription factor matrix family, with the AP2 domain structure and ethylene-responsive element-binding, had the highest number of promoter binding sites of all 20 highly upregulated genes in the AM inoculation signature. Floral homeotic protein APETALA 2, a member of P$FLO2 matrix family, has a documented role in the control of flower and seed development (Jofuku et al., 1994). Strong induction of APETALA 2 in developing nodules of *Medicago truncatula* has been observed and suggested as a potential regulator of the symbiotic program (El Yahyaoui et al., 2004). Another enriched transcription factor matrix family was the P$TOEF matrix family that contains the AP2 domain in its structure and is involved in early activation/response (Table 4, Supplementary Table 6). GO analysis showed that these are involved in organ morphogenesis.

P$CAAT was another potential master regulator of the identified AM colonization signature that contains CCAAT-binding family transcription factors. It has been documented that CCAAT-binding family transcription factors are essential for endosymbiosis establishment and development (Diédhiou and Diouf, 2018). Laser microdissection has documented the expression of CAAT-Box transcription factor in AM, correlated with fungal contact and spread (Hogekamp et al., 2011). Two members of this CCAAT-binding family, *NF-YA1a* and *NF-YA1b*, are positive regulators of AM colonization in soybean (Schaarschmidt et al., 2013). Before the present study, most of the known CCAAT-binding family transcription factors had been reported to be involved in nodulation (Marsh et al., 2007; Soyano et al., 2013). Functional genetic studies of symbiotic genes in *Medicago truncatula* indicate a role for a CCAAT-box transcription factor in rhizobial infection (Cousins, 2016). Analytical approaches based on literature mining have suggested association between a number of potential microRNAs (particularly microRNA169 and microRNA156) and microRNA-regulated transcription factors, which may be involved in the coordinated regulation of nitrogen and phosphorous starvation responses in soybean and *NF-YA3* and *NF-YA8* are targets of microRNA169 (Dehcheshmeh, 2013; Chiasson et al., 2014).

A MYB transcription factor belonging to P$MYBL matrix family was also enriched on promoter region of the identified signature of AM colonization. It has been demonstrated that a transcriptional program for arbuscule degeneration during AM symbiosis is regulated by MYB1 (Floss et al., 2017).

At the regulatory level, promoter analysis of co-expressed genes has demonstrated high potential in identifying key enriched transcription factors, finding undiscovered roles of genes (Deihimi et al., 2012), developing the functional genomics catalog of activated transcription factors during a phenomenon (Mahdi et al., 2013; Zinati et al., 2014), and discovery of transcriptional regulatory networks (Bakhtiarizadeh et al., 2013, 2014b). It has been also shown that number and diversity of differential cis-regulatory elements on promoter regions are strong predictors of gene function and level of expression under different conditions (Babgohari et al., 2014; Shamloo-Dashtpagerdi et al., 2015). This has resulted in developing new indicators of gene importance not based on the gene sequence but on the promoter region. In our previous study, we developed a novel pairwise comparison method for *in silico* discovery of statistically significant cis-regulatory elements in eukaryotic promoter regions (Shamloo-Dashtpagerdi et al., 2015).

Transcription factors have interactions with DNA to regulate gene expression in cells (Pomerantz et al., 2015). In future studies, genome-wide mapping of binding sites of the identified transcription factors [GRAS family transcription factor (MTR_1g069725, MTR_2g089100), AP2 domain transcription factor (MTR_6g029180), and CCAAT-binding transcription factors] by CHIP-seq techniques may unravel the cistrome of successful AM colonization in symbiosis establishment.

## Conclusion

In this study, we developed a new approach for reducing heterogenicity between experiments (batch effect) and direct merging meta-analysis by combining meta-analysis, multi-step normalization, and supervised attribute weighting models. We employed this approach to obtain a unified transcriptomic signature of successful AM colonization in roots of *Medicago truncatula*. The genes of identified in the signature, derived by integration of meta-analysis with supervised attribute weighting models, were strongly up-regulated in all AM symbioses and probably correspond to the end targets of the symbiotic programme. The identified meta-genes of successful AM colonization discriminated efficiently between AM inoculated and non-inoculated samples. Furthermore, the developed signature showed high performance in distinguishing AM-colonized roots from non-inoculated ones in an independent RNA-seq experiment. Important protein classes such as the AP2 domain class transcription factor (MTR_6g029180), GRAS family transcription factors (MTR_1g069725 and MTR_2g089100), and cyclin-dependent kinase (MTR_1g098300) were highly upregulated during AM successful colonization. The developed direct merging-based meta-analysis, by combining meta-analysis, multi-step normalization, and supervised attribute weighting models, provides the possibility of data collection from different experiments even when a treatment or a control is missing in one or more of the experiments.

We suggest that the promoters of meta-genes identified in the transcriptomic signature of AM colonization may have the power to unravel key transcription factors as master regulators of AM symbiosis. Analysis of promoter regions of the top upregulated meta-genes in the AM-successful colonization signature in this study identified enriched transcription factor binding sites and led us to possible master regulators that form the transcriptome expression pattern. These included AP2 domain class transcription factors, CCAAT-binding family transcription factors, SEF transcription factors, and response to fungus ASRC transcription factors. Further functional characterization of these transcription factors is needed to understand their precise role in AM symbioses.

This study provides a framework for an improved understanding of the dynamics of successful AM colonization in establishing microsymbionts. It offers a new approach for related investigations into the other symbiosis systems.

## Author contributions

MM-D, EE, and AN designed the research. ME, MT, ZN, RE, MM-D and EE collected and analyzed the data. ME developed the required software. MM-D and EE wrote the paper. All authors have read and approved the manuscript.

### Conflict of interest statement

The authors declare that the research was conducted in the absence of any commercial or financial relationships that could be construed as a potential conflict of interest.

## Supplementary material

Supplementary Table 1 Available expression data on Arbuscular mycorrhiza at NCBI GEO (https://www.ncbi.nlm.nih.gov/geo/) database.

Supplementary Table 2 Mining transcriptome profile of Medicago roots to achieve transcriptomic signature of Arbuscular mycorrhiza (AM) inoculation. Seven feature selection models including RELIEF, UNCERTAINTY, GINI INDEX, CHI SQUARED, RULE, INFO GAIN, and INFO GAIN RATIO evaluated the relevance of 33685 genes as well as Study ID and AM type in discriminating AM inoculated roots from non-inoculated ones. The resulting weights of each feature selection model were normalized into the interval between 0 and 1. The genes announced important by most of the feature selection models (intersection of weighting methods with various statistical backgrounds) with cut-off > 0.95 were assumed as the key distinguishing genes to form the AM inoculation biosignature.

Supplementary Table 3 Genes discriminating Arbuscular mycorrhiza (AM) inoculated roots from non-inoculated ones according to UNCERTAINTY, GINI INDEX, CHI SQUARED, RULE, INFO GAIN, and INFO GAIN RATIO feature selection models (weight higher than 0.95)and also *Z*-value difference of > 0.5 or < −0.5. The resulting weights of each feature selection model were normalized into the interval between 0 and 1.

Supplementary Table 4 Gene ontology classification of transcriptomic signature of Arbuscular mycorrhiza (AM) inoculation. GO approach in terms of Molecular Function, Biological Process, and Cellular Component was used for functional annotation of 73 unregulated genes in AM inoculation that were announced important by most of feature selection models with *Z*-value difference of >0.5.

Supplementary Table 5 The top 20 highly upregulated meta-genes of successful Arbuscular mycorrhiza (AM) colonization transcriptomic signature on roots of *Medicago truncatula*, obtained by integration of meta-analysis and machine learning, were used for promoter analysis and common regulator discovery.

Supplementary Table 6 Transcription factors matrix families that have enriched binding sites on promoter regions of the top 20 upregulated genes during successful Arbuscular mycorrhiza (AM) colonization according to common TFs analysis of MatInspector tool. Transcription factor Matrix similarity was set to >0.95.

Supplementary Table 7 High correspondence between independent RNA-seq data of Arbuscular mycorrhiza (AM) colonization in *Medicago truncatula* and the identified AM colonization signature in this study, derived by integration of meta-analysis and supervised attribute weighting models.

Supplementary Table 8 RNA-seq based differential expression analysis of *Medicago truncatula* response of Arbuscular mycorrhiza (AM) inoculation compared to non-AM inoculation.

Supplementary Figure 1 Pseudo codes for scaling and quartiling normalization approaches, employed in this study. **(A)** Scaling approach. **(B)** Quartiling approach.
