# Supplementary figures and images for: Unified Transcriptomic Signature of Arbuscular Mycorrhiza Colonization in Roots of Medicago truncatula by Integration of Machine Learning, Promoter Analysis, and Direct Merging Meta-Analysis
Source: Front Plant Sci. 2018 Nov 12;9:1550. doi: 10.3389/fpls.2018.01550 (PMC6240842; doi:10.3389/fpls.2018.01550)

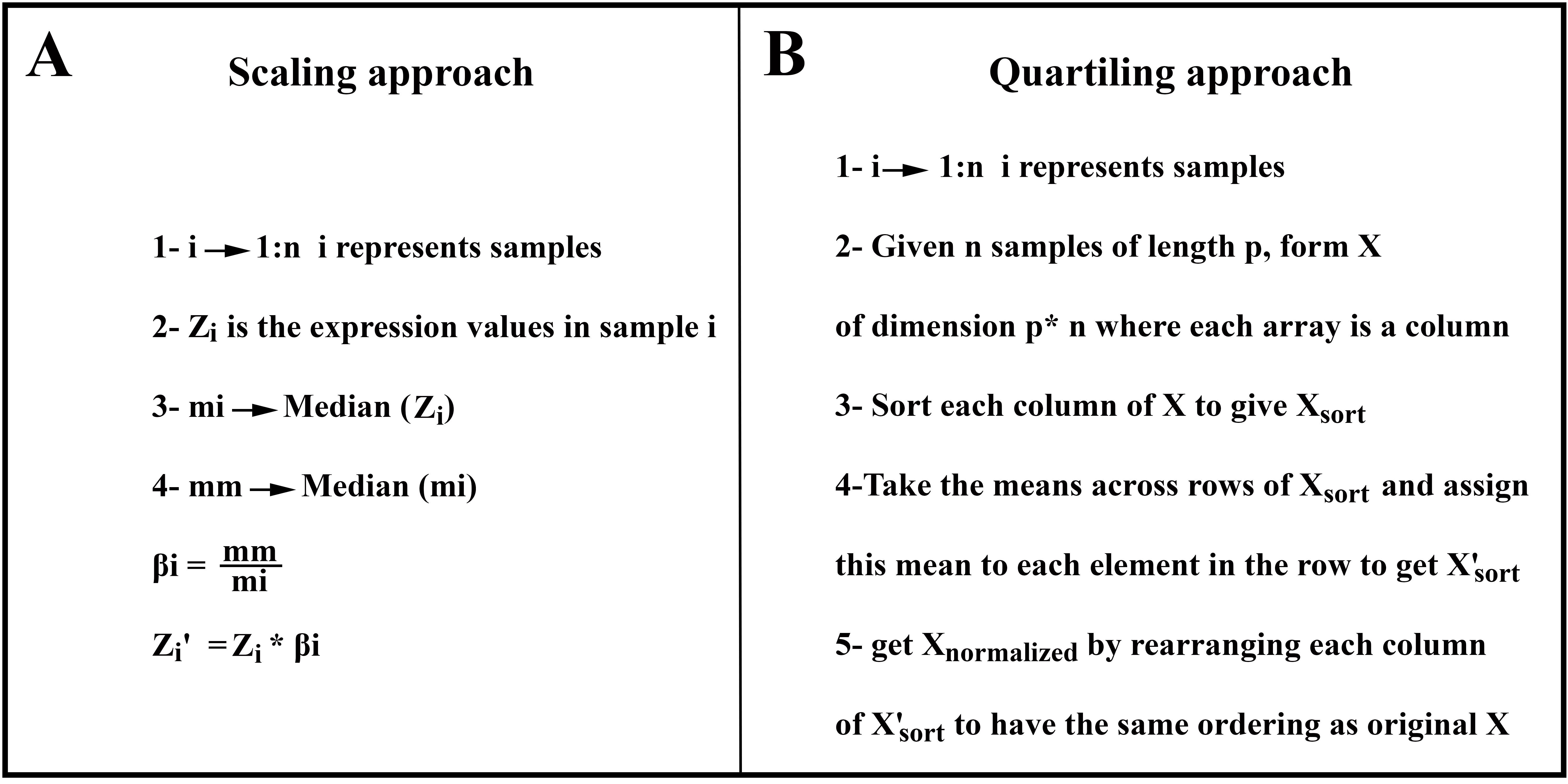

Supplement: Supplementary Figure 1 — Pseudo codes for scaling and quartiling normalization approaches, employed in this study. (A) Scaling approach. (B) Quartiling approach. [file Image_1.TIF]
